# Enhanced Conductivity and Tunable Work Function in VO_X_ Hole-Injection Layers via CsTFSI Doping for Efficient OLEDs

**DOI:** 10.3390/nano16211443

**Published:** 2026-11-07

**Authors:** Zhiyin Feng, Wenqing Zhu, Qidi Lin, Ming Wu, Lu Huang

**Affiliations:** 1 School of Materials Science and Engineering, Shanghai University, 99 Shangda Road, Shanghai 200444, China; iinmutsu@shu.edu.cn (Z.F.); qdlin@shu.edu.cn (Q.L.); mingwu@shu.edu.cn (M.W.); huanglug@126.com (L.H.); 2 Key Laboratory of Advanced Display and System Applications, Ministry of Education, Shanghai University, Yanchang Road 149, Shanghai 200072, China

**Keywords:** vanadium oxide, doping strategy, energy-level alignment, hole-injection layer, organic light-emitting diodes

## Abstract

Balanced charge injection is critical for improving the performance of organic light-emitting diodes (OLEDs). However, hole-injection layer (HIL) materials often suffer from low conductivity and mismatched energy levels, both of which limit hole injection. Herein, we develop an effective strategy using Cesium(I) Bis(trifluoromethanesulfonyl)imide (CsTFSI) to regulate the characteristics of VO_X_ HIL. The energy level alignment and electrical conductivity can be readily tuned by adjusting the doping concentrations of CsTFSI. The optimized device performance is attributed to the synergistic effects of enhanced HIL conductivity and favorable energy level alignment. With fine-tuned HIL, the OLEDs achieved the maximum current efficiency of 67.68 cd A^−1^ and maximum external quantum efficiency of 19.67%, which represent improvements of 23.4% and 26.3% respectively compared to devices with undoped VO_X_ HIL. This work highlights CsTFSI doping as a promising approach for developing high-performance VO_X_ HILs.

## 1. Introduction

Due to the merits of high brightness, wide viewing angle, self-emission and fast response speed, OLEDs have been intensively investigated as a mainstream technology for display and solid-state lighting [1,2]. Despite the great progress in device performance, further efficiency improvement remains essential for broader applications and commercialization. However, inefficient hole injection caused by the large energy barrier at the ITO/HTL interface leads to carrier imbalance and hence limits device performance [3,4]. Consequently, there is an urgent impetus to accelerate the hole injection in OLEDs. HILs have been applied to facilitate hole injection by reducing the interfacial energy barrier [5,6,7,8,9,10,11,12,13,14].

Poly(3,4-ethylenedioxythiophene):poly(styrene-sulfonate) (PEDOT:PSS) is a popular HIL owing to its excellent transparency, good conductivity and suitable work function. However, its acidic and hygroscopic properties would compromise the device stability [15]. As an alternative to PEDOT:PSS, transition metal oxides (TMOs) have gained considerable attention because of their outstanding stability and adjustable energy levels, such as nickel oxide (NiO_X_) [16,17,18], tungsten oxide (WO_3_) [19,20,21], molybdenum oxide (MoO_X_) [22,23,24,25] and vanadium oxide (VO_X_) [26,27,28].

Among various TMOs, VO_X_ has emerged as a competitive HIL material, featuring high optical transparency across the visible range and robust environmental stability [26,27,28]. Notwithstanding these merits, VO_X_ suffers from poor intrinsic conductivity and mismatched energy levels, which restrict hole transport and injection [29,30,31]. Therefore, metal ion doping is proven to be an efficacious approach to ameliorate these characteristics of VO_X_. Much work so far has focused on Li, Cs and other salt modifications, which can elevate the conductivity and tailor the work function of VO_X_, thereby increasing hole injection. For example, in our previous work, we demonstrated that Li doping enhanced the conductivity of VO_X_ by 2.18-fold while adjusting its work function to 5.11 eV [32]. Our subsequent Au/Li co-doping study also simultaneously improved conductivity and energy level alignment [33], whereas the present work achieves a larger relative EQE enhancement over its corresponding undoped control than either of the previous works. Notably, conventional Cs-salt doping reported by Li et al. [34] and Yao et al. [35] mainly regulates the energy-level of VO_X_, yet has not been shown to simultaneously attain a substantial enhancement in conductivity, with high annealing temperature (>200 °C) restricting its applicability in flexible OLED fabrication. Hence, developing a novel doping strategy which can simultaneously elevate the conductivity of VO_X_ and optimize its interfacial energy level alignment is crucial for promoting hole injection and improving OLED efficiency.

In this work, we focus on the comprehensive strategy to simultaneously tailor the energy level and conductivity. Specifically, Cesium(I) Bis(trifluoromethanesulfonyl)imide (CsTFSI) doped VO_X_ was introduced as an outstanding HIL for OLEDs. TFSI-based salts possess advantageous characteristics, including thermal stability, excellent chemical tunability, and high conductivity [36,37,38]. In particular, the strongly electron-withdrawing TFSI^−^ anion has the potential to modulate the energy level of VO_X_ by inducing interfacial dipoles [39]. In addition, CsTFSI modification has been reported to passivate trap states while improving the electrical conductivity and energy level alignment of SnO_2_ [40]. We introduced CsTFSI into VO_X_ with an optimal doping ratio, which increased film conductivity to approximately three times that of pristine VO_X_ and precisely tuned the energy level alignment. Consequently, the hole injection is facilitated, which is helpful to enhance the carrier balance. We found that the device performance is governed by the synergistic effects of HIL conductivity and energy level alignment. As a result, the OLED based on the 3% Cs: VO_X_ HIL exhibits a peak current efficiency of 67.68 cd A^−1^ and an external quantum efficiency of 19.67%, representing a significant boost over the device using pristine VO_X_. These findings establish CsTFSI-doped VO_X_ as a competitive HIL for efficient OLEDs.

## 2. Materials and Methods

Materials: Vanadium triisopropoxide oxide (98%) was purchased from Sigma-Aldrich (St.Louis, MO, USA). Cesium(I) bis(trifluoromethanesulfonyl)imide (CsTFSI, 98%) is obtained from Aladdin (Shanghai, China). 4,4′-cyclohexylidenebis[N,N-bis(p-tolyl)aniline] (TAPC), 1,3-bis(N-carbazolyl)benzene (mCP), tris(2-phenylpyridine)iridium(III) (Ir(ppy)3), 1,3,5-tris(1-phenyl-1H-benzimidazol-2-yl)benzene (TPBi) and 8-hydroxyquinolinolato-lithium (Liq) were purchased from Luminescence Technology Corporation (Taiwan). All materials and reagents were used as received without further purification.

Preparation of VO_X_ and doped VO_X_ precursor solutions: The pristine VO_X_ precursor solution was prepared by mixing 20 μL of vanadium triisopropoxide oxide with 2 mL of anhydrous isopropanol. CsTFSI was then added to the VO_X_ precursor to obtain molar ratios (Cs to V) of 1%, 2%, 3%, and 4%. All mixtures were magnetically stirred at room temperature for 2 h and filtered through a 0.45-μm PTFE syringe filter before use.

Device fabrication: Patterned indium tin oxide (ITO) glass substrates (sheet resistance ~15 Ω sq^−1^) were sequentially cleaned in an ultrasonic bath with detergent, deionized water, acetone, and isopropanol. After drying, the ITO substrates were subjected to a UV-ozone treatment for 15 min. To deposit the HILs, the cleaned ITO substrates were spin-coated with the corresponding pristine or Cs: VO_X_ precursor solutions at 6000 rpm for 60 s. The films were then annealed in ambient air at 130 °C for 20 min. Atmospheric moisture promotes hydrolysis of the vanadium alkoxide precursor, followed by condensation to form the VO_X_ network, while annealing facilitates solvent evaporation and removal of volatile by-products. After annealing, the films underwent a second UV–ozone treatment for 5 min to remove residual organic species and activate the surface before deposition of the subsequent functional layer. Thereafter, the substrates were transferred into a vacuum chamber immediately. The remaining functional layers and the metal cathode were sequentially deposited via vacuum thermal evaporation at a base pressure of <6 × 10^−4^ Pa. The overall multilayer architecture of the fabricated OLEDs was ITO/x mol% Cs: VO_X_ (20 nm)/TAPC (50 nm)/mCP: 6 wt% Ir(ppy)_3_ (35 nm)/TPBi (55 nm)/Liq (1.5 nm)/Al (150 nm), where x is 0, 1, 2, 3, or 4. Within this structure, TAPC served as the hole-transport layer (HTL), mCP doped with the green phosphorescent emitter Ir(ppy)_3_ acted as the emitting layer (EML), TPBi as the electron-transport layer (ETL), and Liq as the electron-injection layer (EIL). The active emission area of all devices was 5 mm^2^, defined by a metal mask.

Characterization: The current density–voltage–luminance (J-V-L) characteristics of the fabricated OLEDs were recorded using a Keithley 2400 SourceMeter (SMU, TEKTRONIX, Solon, OH, USA) coupled with a Photo Research PR-650 SpectraScan spectroradiometer (Spectroradiometer, Photo Research, Syracuse, NY, USA). The turn-on voltage (V_on_) was defined as the voltage at which the luminance reached 1 cd m^−2^. The film thickness, surface morphology, and optical transmittance of the pristine and doped VO_X_ films were characterized using a surface profiler (AlphaStep IQ; KLA-Tencor, Milpitas, CA, USA), an atomic force microscope (AFM, Dimension Icon; Bruker, Santa Barbara, CA, USA), and a UV-visible spectrophotometer (U-3900H; Hitachi, Tokyo, Japan), respectively. X-ray photoelectron spectroscopy (XPS) and ultraviolet photoelectron spectroscopy (UPS) measurements were conducted on a Thermo Scientific ESCALAB 250Xi system. The XPS and UPS spectra were acquired using a monochromatized Al Kα source (1486.7 eV) and a He Iα excitation line (21.22 eV), respectively. The lifetime of devices was measured by an OLED aging measurement system.

## 3. Results and Discussion

### 3.1. Properties of the HIL Films

**Figure 1:**
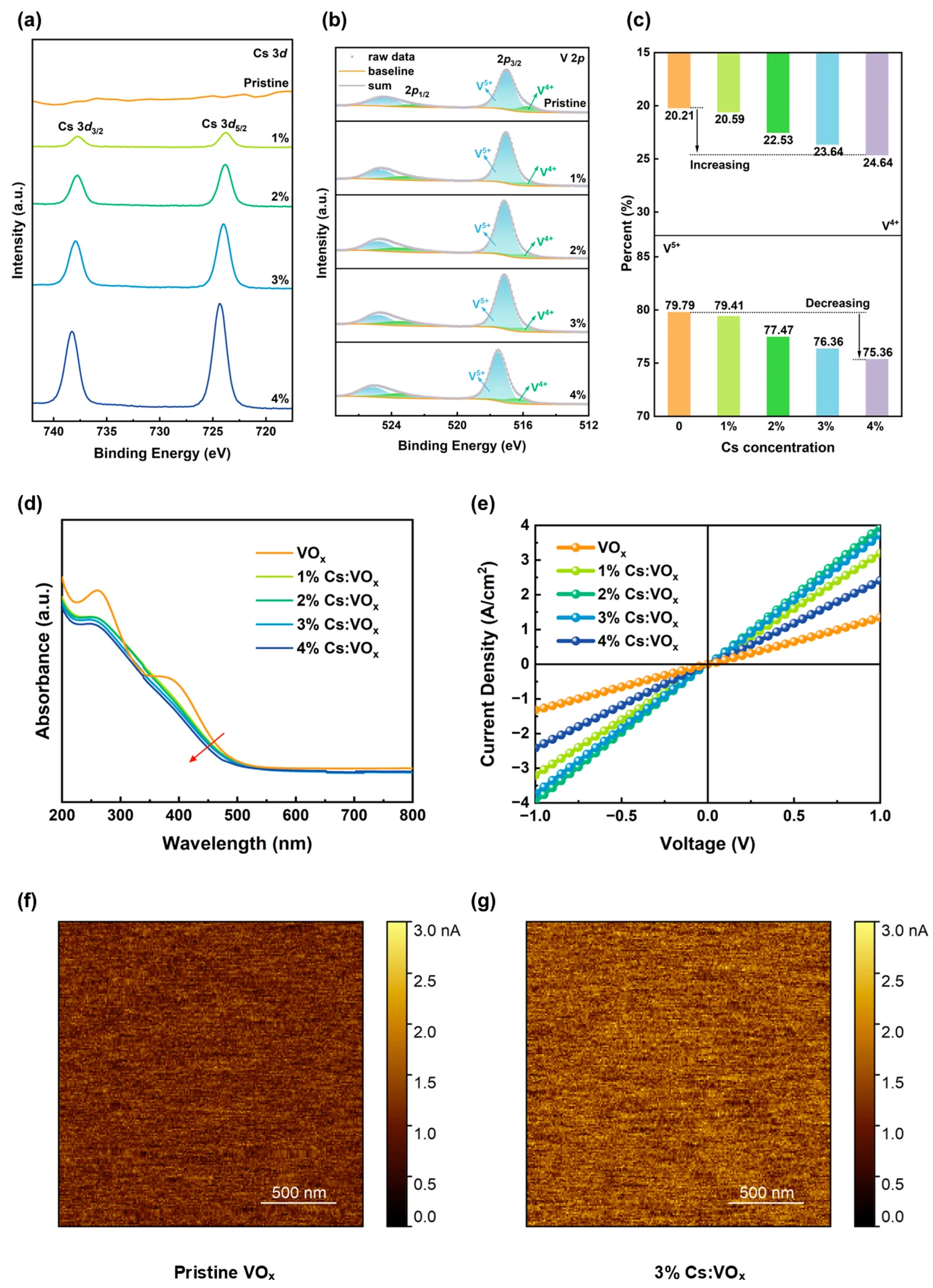
Characterization of pristine and CsTFSI-doped VO_X_ films. (**a**) Cs 3d and (**b**) V 2p XPS spectra. (**c**) V^5+^ and V^4+^ fractions obtained from V 2p fitting. (**d**) UV-vis absorption spectra. (**e**) Current density-voltage curves of ITO/VO_X_/Al and ITO/Cs: VO_X_/Al structures. (**f**,**g**) C-AFM current maps of pristine VO_X_ and 3% Cs: VO_X_, respectively.

X-ray photoelectron spectroscopy (XPS) was conducted to analyze the chemical state of the CsTFSI-doped VO_X_ films, and the spectra were calibrated using C 1s peak at 284.8 eV. The Cs 3d spectrum in Figure 1a displays two distinct peaks near 724 eV and 738 eV, assigned to Cs 3d_5/2_ and Cs 3d_3/2_, respectively, manifesting that Cs is successfully incorporated into the VO_X_ matrix [34]. The F 1s and S 2p spectra further confirm the presence of TFSI-related species in the annealed CsTFSI-modified VO_X_ films (Figures S1 and S2). The V 2p spectra in Figure 1b exhibit a gradual shift toward higher binding energy with increasing CsTFSI content, which can be attributed to the enhanced local positive charge surrounding V atoms [40]. In addition, Figure 1c illustrates that the relative fraction of V^4+^ increases progressively with increasing CsTFSI-doping ratios, indicating a reduction in the average vanadium valence state. As reported in previous studies, an increased V^4+^ proportion favors charge transport in VO_X_, consistent with the observed conductivity improvement [2,41]. O 1s spectra was divided into two peaks, assigned to lattice oxygen (O_L_) and a higher binding energy component tentatively associated with defect-related oxygen environment as shown in Figure S3. For convenience, the latter is denoted as O_V_ in Figure S3 and Table S1. The defect-related fraction initially decreases then rises, with the increase in CsTFSI content (Table S1). Generally, the reduction of V^5+^ to V^4+^ is accompanied by the formation of oxygen vacancies. However, the initial decrease in the high binding energy component of O 1s suggests that moderate CsTFSI incorporation may passivate the related defect, which can impair carrier transport capability [39,42]. Thus, changes in vanadium valence and the oxygen-related defect environment may jointly contribute to the enhanced conductivity of the CsTFSI-doped VO_X_ films.

The optical transmittance spectra in Figure S4 present that CsTFSI doping has little influence on the film transparency. All the films on quartz exhibit high transmittance across the visible spectra region, exceeding 98% at wavelengths above 500 nm when referenced to bare quartz. Furthermore, the Cs: VO_X_ films displayed a blueshift absorption edge (Figure 1d), suggesting a widening of the optical bandgap. Accordingly, the high transparency of these films minimizes the optical losses of the devices, and the films are suitable for application in bottom-emitting OLEDs.

**Figure 2:**
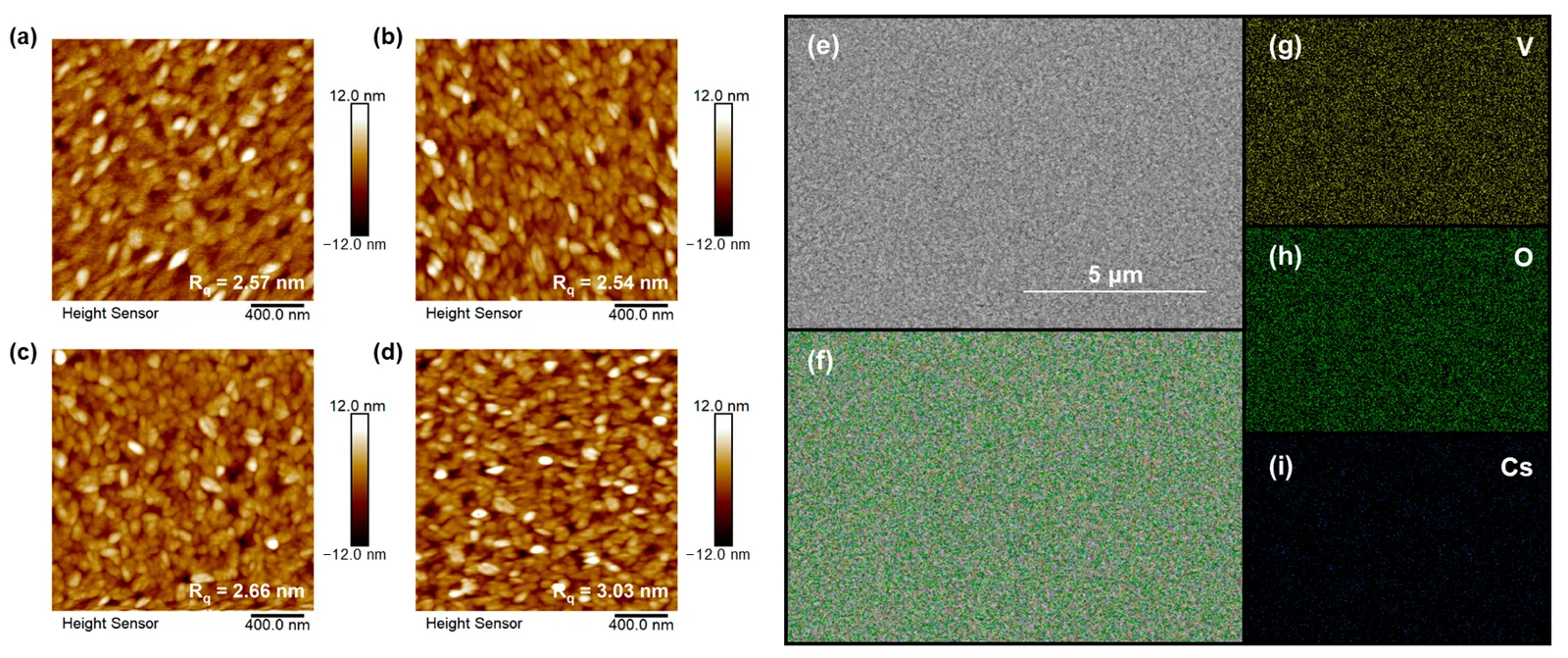
Surface morphology and elemental distribution of Cs: VO_X_ films. (**a**–**d**) AFM height images of 1%, 2%, 3% and 4% Cs: VO_X_, respectively; Rq denotes root-mean-square roughness. (**e**) SEM image of 3% Cs: VO_X_. (**f**) Overlaid EDS map and (**g**–**i**) V, O and Cs maps of the same film, respectively.

To investigate the influence of CsTFSI doping on the surface morphology of VO_X_ films, atomic force microscopy (AFM) and scanning electron microscopy (SEM) characterization was performed as illustrated in Figure 2a–d and Figure S5. The Cs: VO_X_ films with CsTFSI doping ratios of 1–3% exhibited slightly reduced surface roughness compared with 2.71 nm for the undoped film, whereas the roughness increased to 3.03 nm at 4% doping ratio. Consistently, the SEM images in Figure S5 display negligible differences in surface morphologies, with all the films remaining uniform and smooth. These results indicate that moderate CsTFSI doping has little effect on the surface roughness of the films. Furthermore, SEM-energy dispersive X-ray spectroscopy (SEM-EDS) elemental mapping of the 3% Cs: VO_X_ film in Figure 2e–i exhibits homogeneous distributions of V and O across the film, and confirms the presence of Cs. [43]. The contact angle (CA) measurement was used to demonstrate the surface hydrophilicity of VO_X_. It reveals that the Cs: VO_X_ films exhibit high hydrophilicity at lower doping ratios, although the water contact angle gradually rises with higher CsTFSI doping ratio as presented in Figure S6.

In order to explore the effect of CsTFSI doping on the electrical conductivity of VO_X_ films, the apparent conductivity (σ) of the films was calculated based on the current-voltage (I–V) characteristics of the ITO/HIL/Al structure using the equation σ = L/(RS), where L is the film thickness, R is the film resistance obtained from the I–V curve (Figure 1e), and S is the effective area (0.05 cm^2^). The VO_X_ films used for the I–V measurements had a thickness of 20 nm, and the effective electrode overlap area was 0.05 cm^2^. The conductivity increased with the CsTFSI doping ratio, and reached a maximum of 8.07 × 10^−4^ S m^−1^ for the 2% CsTFSI-doped film, approximately three times higher than that of the undoped VO_X_ film. The measured conductivity values may include contributions from the electrode resistance and interfacial contact resistance. By comparison, Yao et al. [35] reported an increase from 7.93 × 10^−4^ S m^−1^ to 1.08 × 10^−3^ S m^−1^, reaching approximately 1.36 times the pristine film. Their films were prepared from aqueous ammonium metavanadate/CsOH·H_2_O and annealed at 210 °C for 8 min, whereas our films were prepared using vanadium triisopropoxide oxide/CsTFSI in isopropanol and were annealed at 130 °C for 20 min. Although the absolute conductivity here was lower, the relative increase over pristine film was greater (approximately 3-fold) and was achieved at a lower annealing temperature. The conductivity improvement can be ascribed to the reduced average vanadium valence state, as evidenced by the increased V^4+^ fraction in the XPS spectra [44]. The conductive atomic force microscopy (C-AFM) images in Figure 1f,g also clearly show the different current levels between undoped and 3% Cs: VO_X_. Considerably increased vertical current is observed in the Cs: VO_X_ film, indicating improved electrical conductivity upon CsTFSI doping. Moreover, the doped film showed uniform current in surface mapping, which is beneficial for more homogeneous charge transport within the VO_X_ film. However, the conductivity decreased when the CsTFSI concentration exceeded 2%, which may due to lattice distortions or defect sites reducing charge carrier mobility.

### 3.2. OLED Device Performance

**Figure 3:**
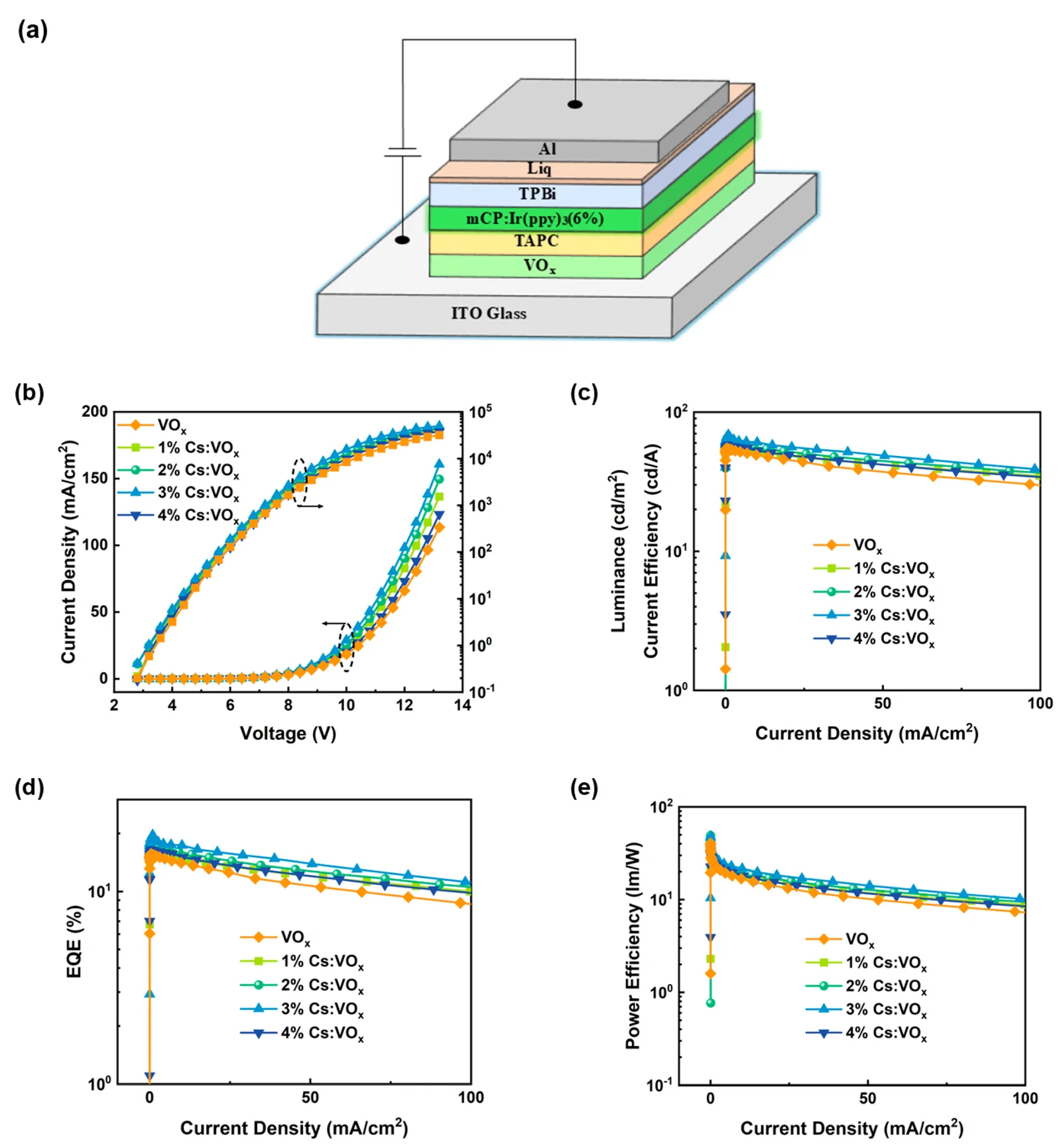
Electroluminescent characteristics of OLEDs with pristine VO_X_ or 1–4% Cs: VO_X_ HILs. (**a**) Schematic of OLED structure. (**b**) Current density-voltage-luminance curves. (**c**) Current efficiency, (**d**) external quantum efficiency (EQE) and (**e**) power efficiency versus current density.

To evaluate the effect of CsTFSI doping on the device performance, the OLEDs were fabricated with the configuration of ITO/HIL/HTL/EML/ETL/Al, as shown in Figure 3a. The performance of devices employing Cs:VO_X_ HILs with different doping concentrations is depicted in Figure 3, and key parameters are summarized in Table 1. The device with 3% Cs:VO_X_ HIL exhibited the highest CE and EQE of 67.68 ± 2.80 cd A^−1^ and 19.67 ± 0.81% based on three independently fabricated devices, corresponding to improvements of 23.4% and 26.3% respectively compared with the reference device based on undoped VO_X_. However, the maximum efficiency is obtained at a 3% doping ratio despite the higher conductivity at 2%, indicating that the improvement cannot be merely attributed to the increase in conductivity. The devices exhibit similar EL spectra and CIE coordinates, as shown in Figure S7.

### 3.3. Interfacial Energy-Level Alignment

**Figure 4:**
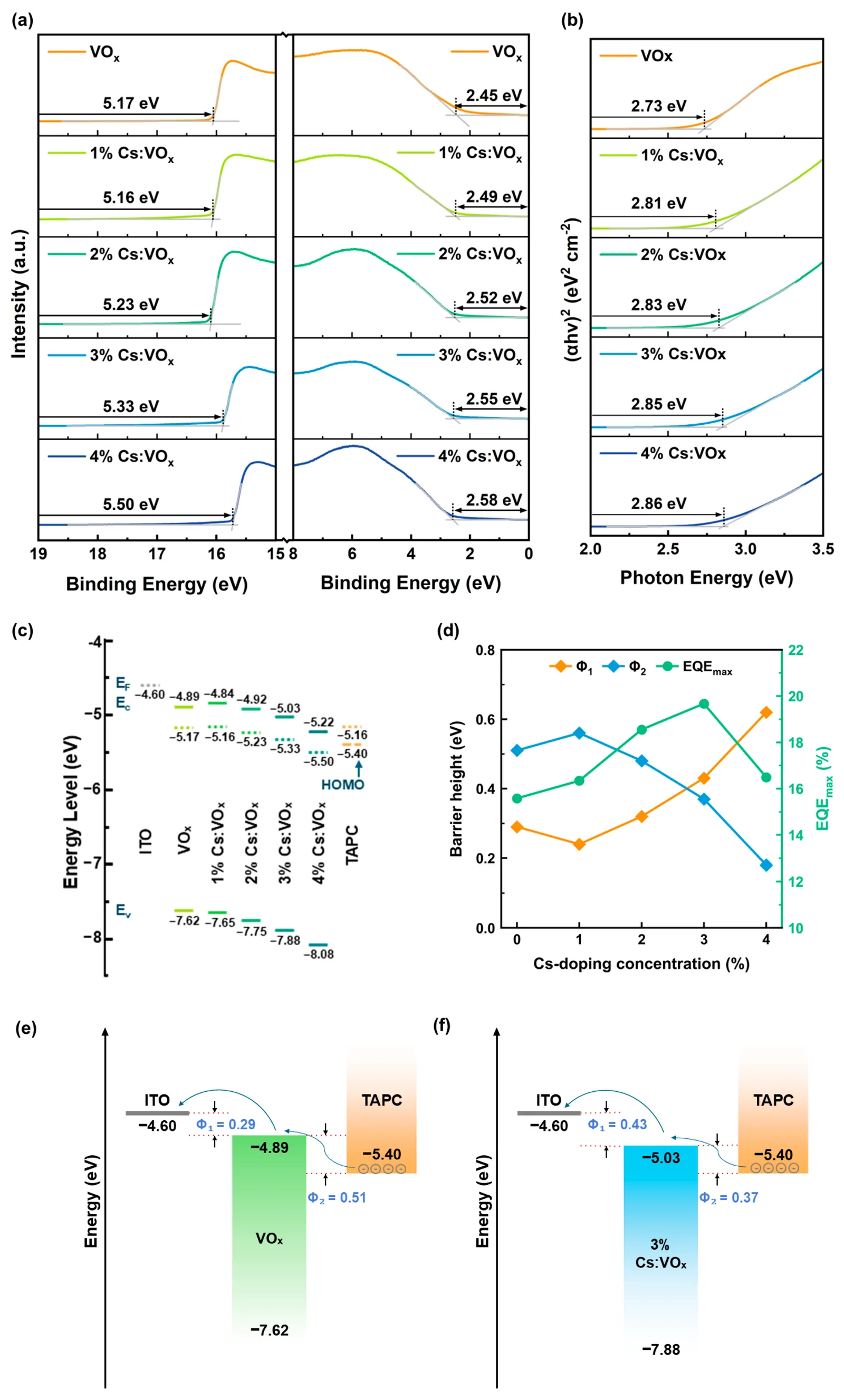
Energy levels and interfacial alignment of pristine and Cs:VO_X_ films. (**a**) UPS secondary-electron cutoff (left) and valence-band (right) spectra. (**b**) Tauc plots. (**c**) Energy levels of ITO, VO_X_, Cs:VO_X_ and TAPC relative to vacuum; EF, EC and EV denote the Fermi level, conduction-band minimum and valence-band maximum, respectively. (**d**) Sub-barrier heights at the ITO/HIL (Φ_1_) and HIL/TAPC (Φ_2_) interfaces, and maximum EQE versus CsTFSI doping ratio. (**e**,**f**) ITO/HIL/TAPC energy-level diagrams for pristine VO_X_ and 3% Cs:VO_X_, respectively. All energies are in eV.

To clearly elucidate the mechanism for the device improvement, ultraviolet photoelectron spectroscopy (UPS) measurements were performed on each film with various doping concentration. As shown in Figure 4a, the work function of the VO_X_ films increases steadily with the CsTFSI-doping ratio, rising from approximately 5.17 eV for the undoped film to about 5.50 eV at 4% CsTFSI doping. This pronounced rise in work function stems from the reduced V^5+^/V^4+^ ratio (Figure 1c), which induces a downward shift in the Fermi level (EF) with increasing CsTFSI incorporation [27]. Valence-band and optical bandgap analyses (Figure 4a,b) reveal that CsTFSI doping downshifts both the VBM and CBM of VO_X_, with detailed energy-level parameters and calculation process summarized in Table S2. Although the work function increases from 5.17 to 5.50 eV, the derived E_C_-E_F_ remains nearly constant at 0.28–0.32 eV, indicating that the work-function variation mainly reflects a shift in E_F_ relative to the vacuum level rather than a substantial change in its position within the bandgap. This behavior may also be associated with the changes in the surface dipole induced by CsTFSI incorporation. In this work, the CBM was estimated by combining the UPS-derived VBM with the optical bandgap. Subsequently, we compared the energy level alignment at anode interface across doping concentrations, as illustrated in Figure 4c,e,f. Hole injection is proposed to proceed via electron extraction from the TAPC HOMO into the VO_X_ conduction band, followed by electron transport to ITO, leaving holes in TAPC. A Cs:VO_X_ HIL serves to divide the large initial energy barrier between ITO and TAPC into two smaller ones: the barrier Φ_1_ at ITO/Cs:VO_X_ and Φ_2_ at Cs:VO_X_/TAPC. The calculated sub-barrier heights and corresponding EQE for devices employing a series of Cs:VO_X_ HILs are illustrated in Figure 4d. With increasing CsTFSI concentration, Φ_1_ become gradually closer to Φ_2_, demonstrating that energy levels are continuously regulated. At 3% doping ratio, the two sub-barriers are 0.43 and 0.37 eV, respectively, accompanied by the highest EQE. However, although pristine VO_X_ does not have the poorest energy level matching, its low conductivity may limit its efficiency, suggesting synergistic effects of conductivity and energy-level alignment.

### 3.4. Charge Transport Dynamics and Stability

**Figure 5:**
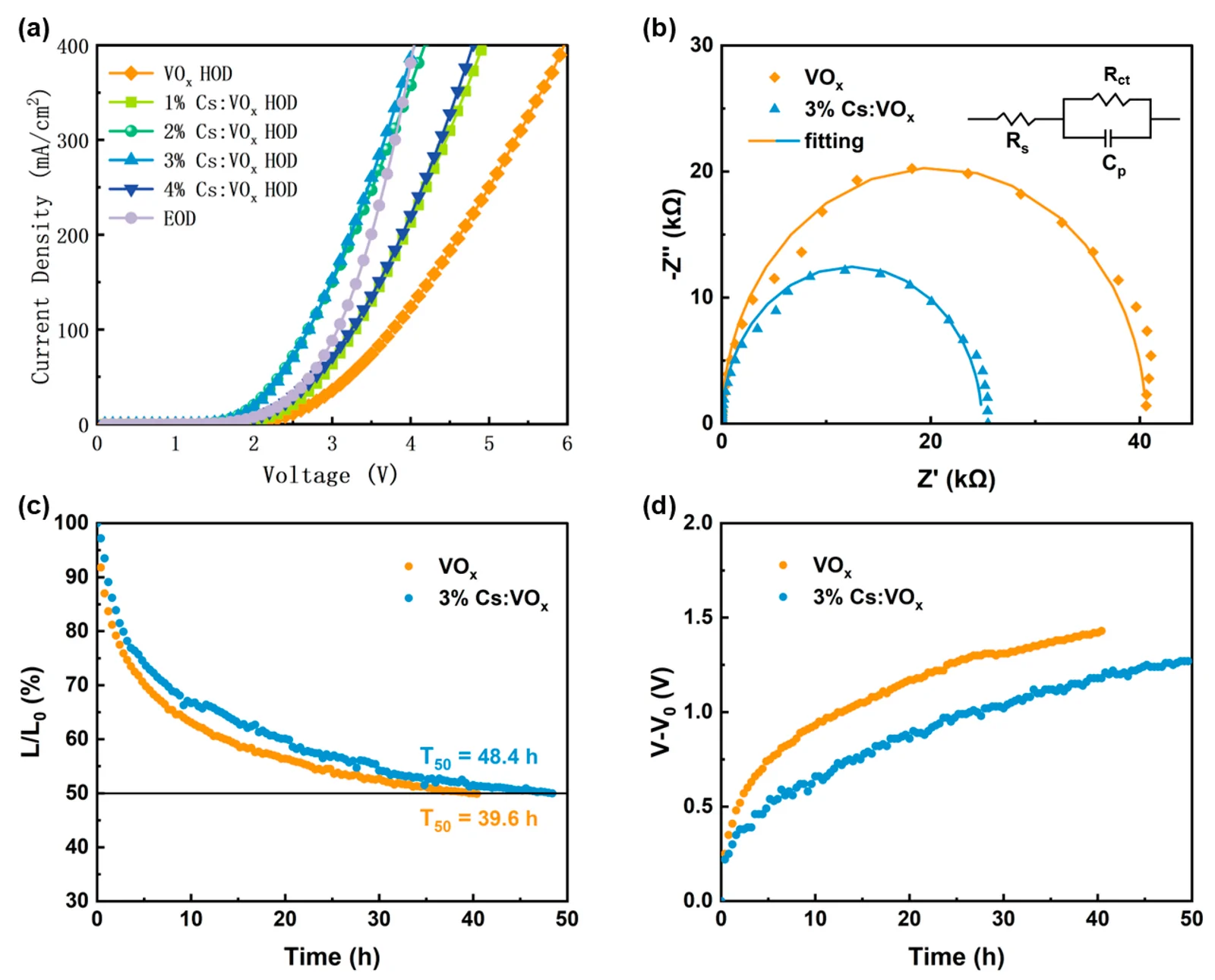
Charge transport and operational stability. (**a**) Current density-voltage curves of HODs with pristine VO_X_ or 1–4% Cs:VO_X_ HILs and the reference EOD. (**b**) Nyquist plots and fits for HODs with pristine VO_X_ and 3% Cs:VO_X_; inset, equivalent circuit. (**c**) Normalized luminance (L/L_0_) and (**d**) voltage increase (V-V_0_) of OLEDs with pristine VO_X_ and 3% Cs:VO_X_ HILs under constant current density. L_0_ and V_0_ are the initial luminance and voltage, respectively.

To gain more insight into the energy level structure of the hole injection process, hole-only devices (HODs) with the structure ITO/x mol% Cs:VO_X_/TAPC (50 nm)/MoO_3_ (5 nm)/Al (150 nm) (where x = 0, 1, 2, 3, or 4) were fabricated. An electron-only device (EOD) with the structure ITO/TPBi (55 nm)/Liq (1.5 nm)/Al (150 nm) was used as a reference. As a result, the J-V characteristics of these single-carrier devices are presented in Figure 5a. The HODs based on CsTFSI doping HILs exhibit larger current densities at the same applied voltage, indicating an enhanced hole injection. Furthermore, the hole currents become much closer to the electron current of the EOD, suggesting that the carrier injection tended to be balanced. Specifically, the highest current density was achieved using 3% Cs:VO_X_ with Φ_1_ = 0.43 eV and Φ_2_ = 0.37 eV, which are closest among several films, alongside relatively high conductivity. These results reveal that the enhanced hole injection originates from the combined effects of improved HIL conductivity and favorable energy level alignment, which is consistent with the above device performance.

To further investigate the interfacial transport mechanism, electrochemical impedance spectroscopy (EIS) was performed. The measurements were conducted over a frequency range of 1 Hz to 1 MHz at a bias of 3 V with an AC amplitude of 10 mV. Figure 5b presents the Nyquist plots of the HODs with different HILs, and it shows that the arc radius of the 3% Cs:VO_X_HOD is smaller than that of the pristine HOD, demonstrating that the doped VO_X_ has a lower transfer resistance. The spectra were fitted using the equivalent circuit consisting of R_s_ and R_ct_ (R_s_ represents the series resistance and R_ct_ denotes the charge transfer resistance), as presented in the inset of Figure 5b. The optimized circuit parameters are summarized in Table S3. Compared with the device based on pristine VO_X_, the 3% Cs:VO_X_ HOD exhibits a clear reduction in R_s_ from 84.07 to 52.22 Ω and in R_ct_ from 40.59 to 24.90 kΩ. The significantly reduced R_s_ and R_ct_ can be attributed to the enhanced charge transfer property arising from the combined effects of improved conductivity and favorable energy level alignment in the 3% Cs:VO_X_ HOD [45,46]. Consequently, the HOD and EIS measurements together clearly corroborate the superior hole injection and interfacial charge transfer characteristics of the 3% Cs:VO_X_ device, in good agreement with the synergistic interpretation.

The evaluation of operational stability is crucial for practical deployment of OLEDs. To assess the influence of CsTFSI-doped VO_X_ HIL on device durability, the operational lifetime was measured under constant current density of 2 mA cm^−2^ in air at room temperature (40% relative humidity). Three independently fabricated, unencapsulated devices were tested for each HIL type. As plotted in Figure 5c, the 3% Cs:VO_X_ based device shows a longer T_50_ lifetime (time to 50% of the initial luminance) of 48.4 h compared to 39.6 h for the undoped VO_X_ based device. Furthermore, the device utilizing the 3% Cs:VO_X_ layer displayed a slower rise in operating voltage during continuous operation, as illustrated in Figure 5d, indicating the interfacial stability is elevated on the Cs:VO_X_ HIL [47].

## 4. Conclusions

In this work, we demonstrated that regulating both the work function and conductivity of VO_X_ through CsTFSI doping is a successful strategy to improve the performance of Ir(ppy)_3_-based OLEDs. Through CsTFSI doping, the electrical conductivity and energy level of VO_X_ were simultaneously regulated while maintaining uniform conductivity across the surface. The film conductivity was elevated up to approximately three times of undoped VO_X_, promoting the hole transport through the HIL. With fine-tuned work function, the energy level matching between the anode/HIL and HIL/HTL interfaces were optimized, thereby facilitating hole injection. These synergistical effects enhance carrier balance, resulting in higher device efficiency. As a result, the green phosphorescent OLED employing the 3% Cs:VO_X_ HIL delivered a maximum EQE of 19.67%, representing a 26.3% improvement over the undoped VO_X_ device. These results establish that CsTFSI modification provides a practical route for concurrently tuning bulk charge transport and interfacial energy-level properties in solution-processed VO_X_ HILs.

## Figures and Tables

**Table 1:** Performance parameters of OLED devices based on Cs: VO_X_ films with different CsTFSI doping ratios as HILs. Three devices were measured for each HIL composition, and all values represent the averages of the corresponding parameters obtained from the three devices.

HIL Type	*V*_on_ (V)	CE_max_ (cd A^−1^)	EQE_max_ (%)	PE_max_ (lm W^−1^)	EQE at 1000 cd m^−2^ (%)
VO_X_	3.47	54.85	15.58	40.57	15.29
1% Cs:VO_X_	3.35	57.30	16.34	41.44	15.94
2% Cs:VO_X_	3.24	64.88	18.55	49.06	17.27
3% Cs:VO_X_	3.21	67.68	19.67	48.15	18.67
4% Cs:VO_X_	3.37	58.24	16.49	36.17	16.23

## Data Availability

The original contributions presented in this study are included in the article/Supplementary Material. Further inquiries can be directed to the corresponding author(s).

## Supplementary Materials

The following supporting information can be downloaded at https://www.mdpi.com/article/10.3390/nano16211443/s1, Figure S1: XPS survey spectra of the pristine VO_X_ and annealed 3 mol% CsTFSI-doped VO_X_ films; Figure S2: F 1s and S 2p spectra of the pristine VO_X_ and annealed 3 mol% CsTFSI-doped VO_X_ films; Figure S3: O 1s XPS spectra of Cs:VO_X_ films with different CsTFSI doping ratios; Figure S4: Transmittance of Cs:VO_X_ films with different CsTFSI doping ratios; Figure S5: SEM images of Cs:VO_X_ films with different CsTFSI doping ratios: (a) 1% Cs:VO_X_, (b) 2% Cs:VO_X_, (c) 3% Cs:VO_X_ and (d) 4% Cs:VO_X_; Figure S6: Water contact angles of Cs:VO_X_ films with different CsTFSI doping ratios: (a) 1% Cs:VO_X_, (b) 2% Cs:VO_X_, (c) 3% Cs:VO_X_ and (d) 4% Cs:VO_X_; Figure S7: (a) EL spectra of the devices with different HIL. (b) CIE coordinate of the OLEDs. Table S1: Proportion of oxygen vacancies related component in Cs:VO_X_ films with different CsTFSI doping ratios; Table S2: Summary of the energy-level parameters of VO_X_ and Cs-doped VO_X_ films, including work function (WF), valence band maximum (VBM), bandgap (Eg), and conduction band minimum (CBM), extracted from UPS and Tauc-plot analyses; Table S3: Impedance fitting parameters for HODs based on different HILs.

## Abbreviations

The following abbreviations are used in this manuscript:

HIL	Hole-injection layer
OLEDs	Organic light-emitting diodes
PEDOT:PSS	Poly(3,4-ethylenedioxythiophene):poly(styrene sulfonate)
CsTFSI	Cesium(I) bis(trifluoromethanesulfonyl)imide
XPS	X-ray photoelectron spectroscopy
AFM	Atomic force microscopy
SEM	Scanning electron microscopy
CE	Current efficiency
EQE	External quantum efficiency
UPS	Ultraviolet photoelectron spectroscopy
VBM	Valence band maximum
CBM	Conduction band minimum
HODs	Hole-only devices
EOD	Electron-only devices
EIS	Electrochemical impedance spectroscopy
