# Supplementary material for: Enhanced Conductivity and Tunable Work Function in VOX Hole-Injection Layers via CsTFSI Doping for Efficient OLEDs
Source: Nanomaterials (Basel). 2026 Nov 7;16(21):1443. doi: 10.3390/nano16211443 (PMC13765385; doi:10.3390/nano16211443)
Supplement: Supplementary file 1 [file nanomaterials-16-01443-s001.zip › nanomaterials-4650154-supplementary.pdf]

Electronic Supplementary Information (ESI)

Enhanced Conductivity and Tunable Work Function in VO_x Hole-Injection Layers via CsTFSI Doping for Efficient OLEDs

Zhiyin Feng [ab], *Wenqing Zhu* [*ab], *Qidi Lin* [ab], *Ming Wu* [ab], *Lu Huang* [a]

[a] School of Materials Science and Engineering, Shanghai University, Yanchang Road 149, 200072 Shanghai, People's Republic of China

[b] Key Laboratory of Advanced Display and System Applications, Ministry of Education, Shanghai University, Yanchang Road 149, 200072 Shanghai, People's Republic of China

Corresponding Author:

Wenqing Zhu

Yanchang Road 149, 200072 Shanghai, People's Republic of China

E-mail: wqzhu@shu.edu.cn

Tel: +86-021-56331976

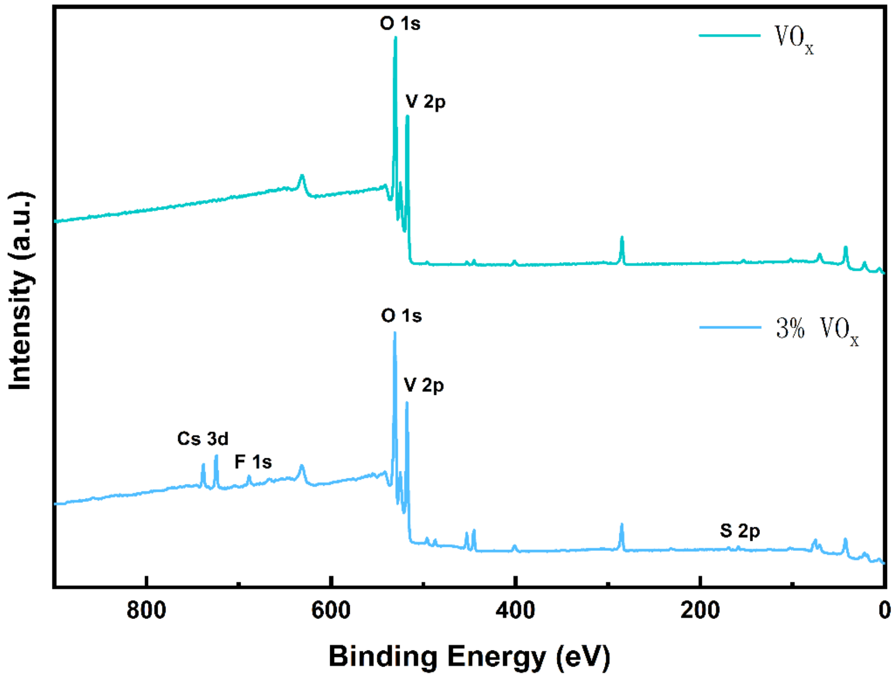

Figure S1 XPS survey spectra of the pristine VO_x and annealed 3 mol% CsTFSI-doped VO_x films.

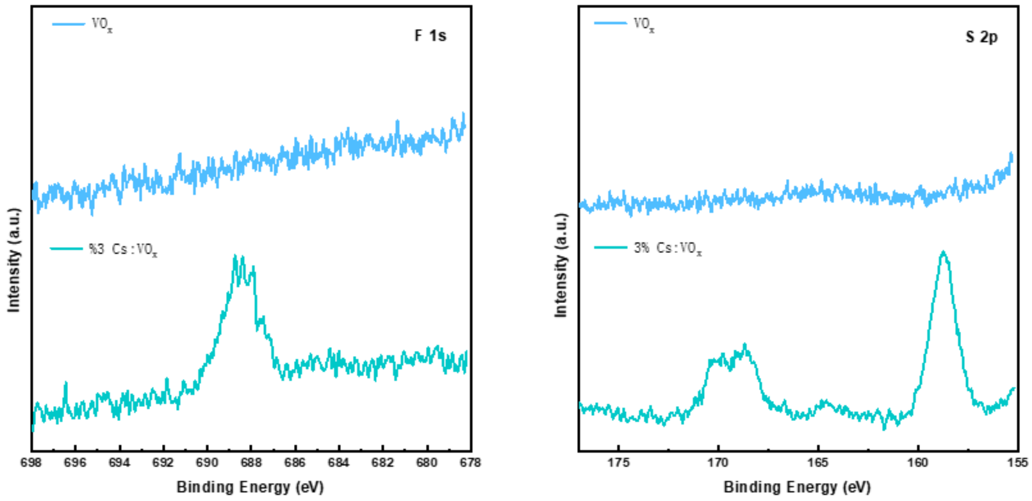

Figure S2 F 1s and S 2p spectra of the pristine VO_x and annealed 3 mol% CsTFSI-doped VO_x films.

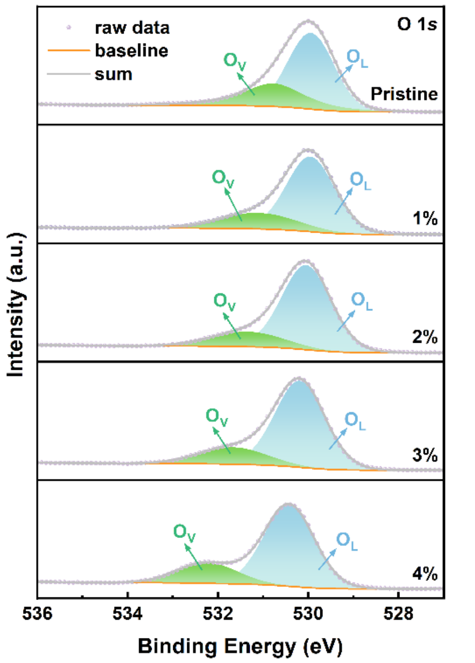

Figure S3 O 1s XPS spectra of Cs:VO_x films with different CsTFSI doping ratios.

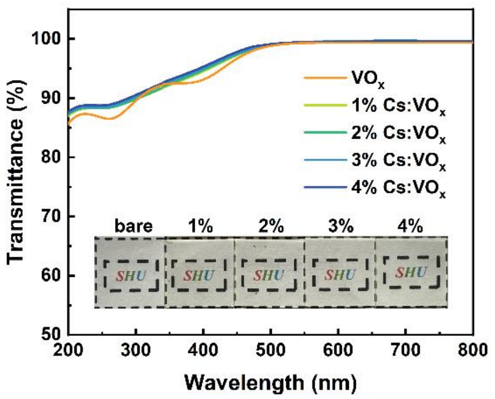

Figure S4 Transmittance of Cs:VO_x films with different CsTFSI doping ratios.

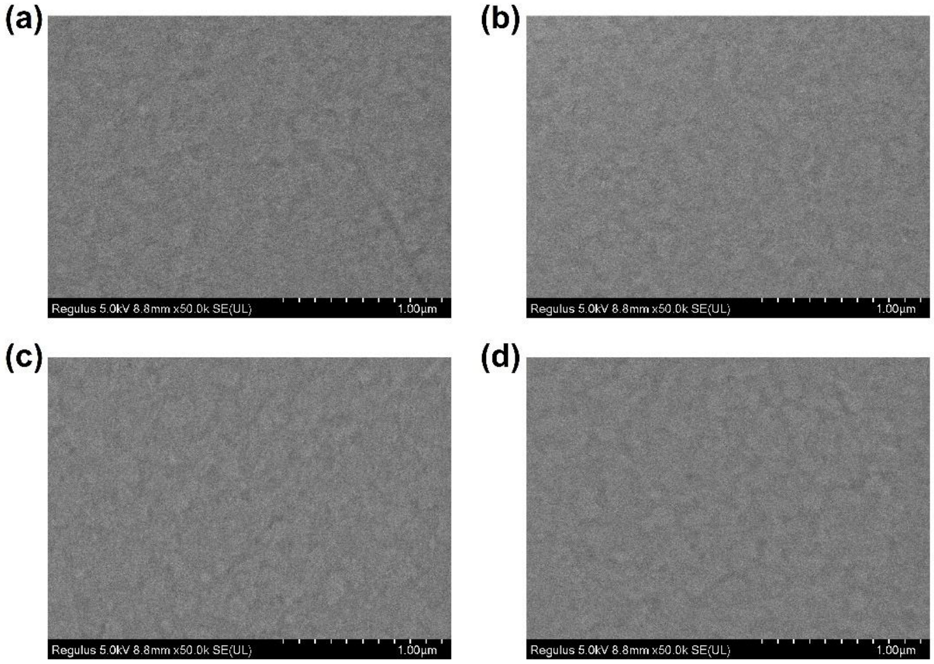

Figure S5 SEM images of Cs:VO_x films with different CsTFSI doping ratios: (a) 1% Cs:VO_x, (b) 2% Cs:VO_x, (c) 3% Cs:VO_x and (d) 4% Cs:VO_x.

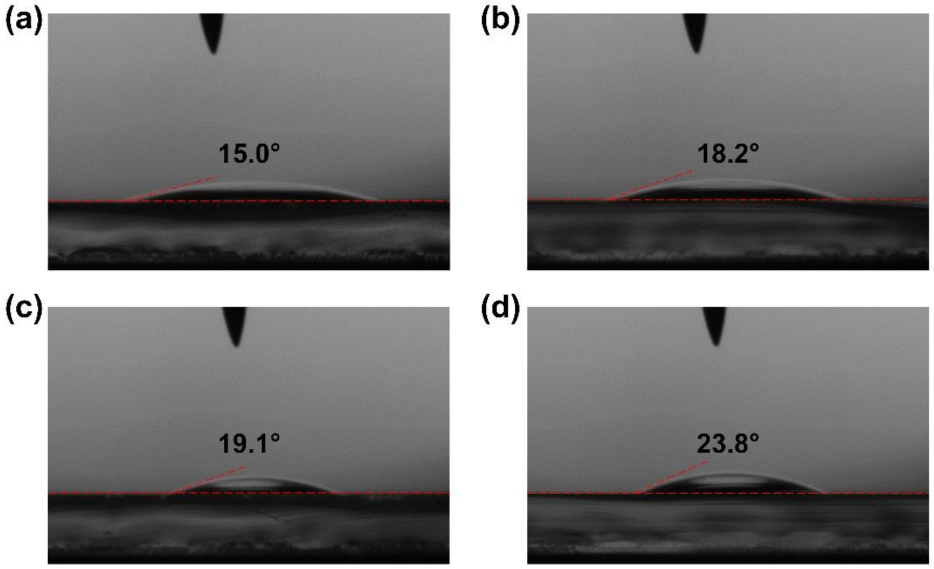

Figure S6 Water contact angles of Cs:VO_x films with different CsTFSI doping ratios: (a) 1% Cs:VO_x, (b) 2% Cs:VO_x, (c) 3% Cs:VO_x and (d) 4% Cs:VO_x.

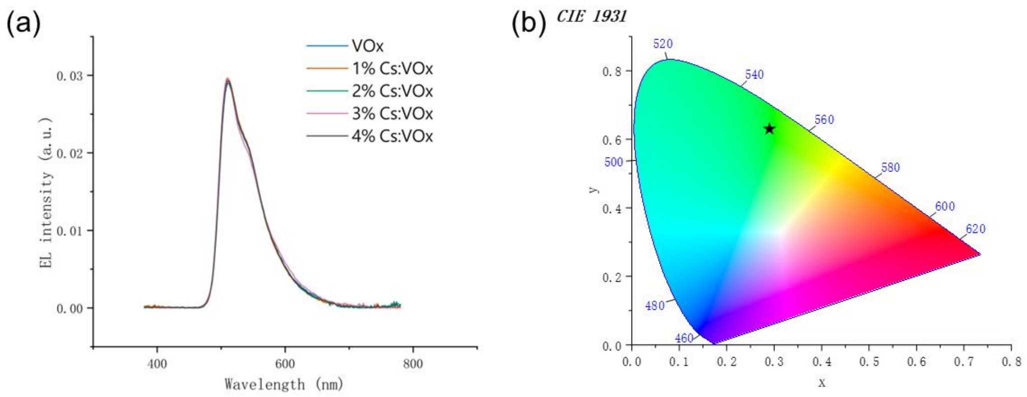

Figure S7 (a) EL spectra of the devices with different HIL. (b) CIE coordinate of the OLEDs.

Table S1 Proportion of oxygen vacancies related component in Cs:VO_x films with different CsTFSI doping ratios

doping ratio (%)	0	1	2	3	4
O_V (%)	31.9	25.2	20.4	20.9	23.0

Table S2 Summary of the energy-level parameters of VO_x and Cs-doped VO_x films, including work function (WF), valence band maximum (VBM), bandgap (Eg), and conduction band minimum (CBM), extracted from UPS and Tauc-plot analyses. WF and VBM were derived from the UPS secondary-electron cutoff and valence-band onset, and CBM was estimated by subtracting the optical bandgap from VBM.

**Doping Ratio**	**Work Function WF (eV)**	**(VBM – E_F) (eV)**	**VBM (eV)**	**Optical Bandgap Eg (eV)**	**CBM = VBM – Eg (eV)**
VO_x	5.17	2.45	7.62	2.73	4.89
1% Cs:VO_x	5.16	2.49	7.65	2.81	4.84
2% Cs:VO_x	5.23	2.52	7.75	2.83	4.92
3% Cs:VO_x	5.33	2.55	7.88	2.85	5.03
4% Cs:VO_x	5.50	2.58	8.08	2.86	5.22

Table S3 Impedance fitting parameters for HODs based on different HILs.

doping ratio (%)	$R_S(\Omega)$	$R_{ct}(k\Omega)$
0	84.07	40.59
1	60.91	30.48
2	50.11	24.75
3	52.22	24.90
4	68.80	34.88
